# Correction: ChatGPT versus human in generating medical graduate exam multiple choice questions—A multinational prospective study (Hong Kong S.A.R., Singapore, Ireland, and the United Kingdom)

**DOI:** 10.1371/journal.pone.0327290

**Published:** 2025-06-27

**Authors:** Billy Ho Hung Cheung, Gary Kui Kai Lau, Gordon Tin Chun Wong, Elaine Yuen Phin Lee, Dhananjay Kulkarni, Choon Sheong Seow, Ruby Wong, Michael Tiong-Hong Co

The Funding statement for this article is incorrect. The correct Funding statement is as follows: This publication was made possible in part by the support from the HKU Libraries Open Access Author Fund sponsored by the HKU Libraries.
