# Supplementary material for: ChatGPT versus human in generating medical graduate exam multiple choice questions—A multinational prospective study (Hong Kong S.A.R., Singapore, Ireland, and the United Kingdom)
Source: PLoS One. 2023 Aug 29;18(8):e0290691. doi: 10.1371/journal.pone.0290691 (PMC10464959; doi:10.1371/journal.pone.0290691)
Supplement: S1 File — (DOCX) [file pone.0290691.s002.docx]

ChatGPT Webpage

User:

Can you write a multiple-choice question based on the following criteria, with the reference I am providing for you and your medical knowledge?

1. The questions were designed to meet the standard for a medical graduate exam.
2. Only four choices were allowed for each question.
3. The questions were limited to knowledge-based questions only.
4. Distractors were allowed to refer from sources other than the original reference provided.
5. An answer is required.
6. No explanation is required for the question.

Reference:

Acute pancreatitis

Incidence

Acute pancreatitis accounts for 3% of all cases of abdominal pain among patients admitted to hospital in the UK. The hospital admission rate for acute pancreatitis is 9.8 per year per 100 000 population in the UK, although worldwide, the annual incidence may range from 5 to 50 per 100 000. The disease may occur at any age, with a peak in young men and older women.

Aetiology

The two major causes of acute pancreatitis are biliary calculi, which occur in 50–70% of patients, and alcohol abuse, which accounts for 25% of cases. Gallstone pancreatitis is thought to be triggered by the passage of gallstones down the common bile duct. If the biliary and pancreatic ducts join to share a common channel before ending at the ampulla, then obstruction of this passage may lead to reflux of bile or activated pancreatic enzymes into the pancreatic duct. Patients who have small gallstones and a wide cystic duct may be at a higher risk of passing stones. The proposed mechanisms for alcoholic pancreatitis include the effects of diet, malnutrition, direct toxicity of alcohol, concomitant tobacco smoking, hypersecretion, duct obstruction or reflux, and hyperlipidaemia. The remaining cases may be due to rare causes or be idiopathic.

Among patients who undergo ERCP, 1–3% develop pancreatitis, probably as a consequence of duct disruption and enzyme extravasation. Patients with sphincter of Oddi dysfunction or a history of recurrent pancreatitis, and those who undergo sphincterotomy or balloon dilatation of the sphincter, carry a higher risk of developing post-ERCP pancreatitis. Patients who have undergone upper abdominal or cardiothoracic surgery may develop acute pancreatitis in the postoperative phase, as may those who have suffered blunt abdominal trauma.

Hereditary pancreatitis is a rare familial condition associated with mutations of the cationic trypsinogen gene. Patients have a tendency to suffer acute pancreatitis while in their teens, progress to chronic pancreatitis in the next two decades and have a high risk (possibly up to 40%) of developing pancreatic cancer by the age of 70 years.

Occasionally, tumours at the ampulla of Vater may cause acute pancreatitis. It is important to check the serum calcium level, a fasting lipid profile, autoimmune markers and viral titres in patients with so called idiopathic acute pancreatitis. It is equally important to take a detailed drug history and remember the association of corticosteroids, azathioprine, asparaginase and valproic acid with acute pancreatitis. Statins (taken over a long time) and gliptins have been linked with pancreatitis, but the evidence is slim. It is essential to exclude tiny gallstones. A careful search for the aetiology must be made in all cases, and no more than 20% of cases should fall into the idiopathic category.

Clinical presentation

Pain is the cardinal symptom. It characteristically develops quickly, reaching maximum intensity within minutes rather than hours and persists for hours or even days. The pain is frequently severe, constant and refractory to the usual doses of analgesics. Pain is usually experienced first in the epigastrium but may be localised to either upper quadrant or felt diffusely throughout the abdomen. There is radiation to the back in about 50% of patients, and some patients may gain relief by sitting or leaning forwards. The suddenness of onset may simulate a perforated peptic ulcer, while biliary colic or acute cholecystitis can be mimicked if the pain is maximal in the right upper quadrant. Radiation to the chest can simulate myocardial infarction, pneumonia or pleuritic pain. In fact, acute pancreatitis can mimic most causes of the acute abdomen and should seldom be discounted in differential diagnosis.

Nausea, repeated vomiting and retching are usually marked accompaniments. The retching may persist despite the stomach being kept empty by nasogastric aspiration. Hiccoughs can be troublesome and may be due to gastric distension or irritation of the diaphragm.

On examination, the appearance may be that of a patient who is well or, at the other extreme, one who is gravely ill with profound shock, toxicity and confusion. Tachypnoea is common, tachycardia is usual, and hypotension may be present. The body temperature is often normal or even subnormal, but frequently rises as inflammation develops. It is useful to reiterate here that SIRS is defined by the presence of two or more of the following criteria: heart rate >90/min, core temperature <36 o C or >38 o C, respirations >20/min or pCO 2 <32 mmHg, and white blood cell count <4000 or >12 000/mm3 . Mild icterus can be caused by biliary obstruction in gallstone pancreatitis, and an acute swinging pyrexia suggests cholangitis. Bleeding into the fascial planes can produce bluish discolouration of the flanks (Grey Turner’s sign) or umbilicus (Cullen’s sign). Neither sign is pathognomonic of acute pancreatitis; Cullen’s sign was first described in association with rupture of an ectopic pregnancy. Subcutaneous fat necrosis may produce small, red, tender nodules on the skin of the legs.

Abdominal examination may reveal distension due to ileus or, more rarely, ascites with shifting dullness. A mass can develop in the epigastrium due to inflammation. There is usually muscle guarding in the upper abdomen, although marked rigidity is unusual. A pleural effusion is present in 10–20% of patients. Pulmonary oedema and pneumonitis are also described and may give rise to the differential diagnosis of pneumonia or myocardial infarction. The patient may be confused and exhibit the signs of metabolic derangement together with hypoxaemia.

Investigations

Typically, the diagnosis is made on the basis of the clinical presentation and an elevated serum amylase level. A serum amylase level three times above normal is indicative of the disease. A normal serum amylase level does not exclude acute pancreatitis, particularly if the patient has presented a few days later. If the serum lipase level can be checked, it provides a slightly more sensitive and specific test than amylase. If there is doubt, and other causes of acute abdomen have to be excluded, contrast-enhanced CT is probably the best single imaging investigation.

ASSESSMENT OF SEVERITY

It is important to define that group of patients who will develop severe pancreatitis, as they are the ones with poorer outcomes and therefore they require aggressive early management and possibly transfer to a specialist unit. A severe attack may be heralded by an initial clinical impression of a very ill patient and a worsening physiological state at 24–48 hours. Various prognostic scoring systems have been used, all aimed at predicting persistent organ failure, particularly respiratory, cardiac and renal. Severity stratification assessments should be performed in patients at 24 hours, 48 hours and 7 days after admission. The Ranson and Glasgow scoring systems are specific for acute pancreatitis, and a score of 3 or more at 48 hours indicates a severe attack (Table 68.3). Several other systems that are used in intensive care units can also be applied. These include the APACHE, SAPS, SOFA, MODS and modified Marshall scoring systems (the latter has the advantage of simplicity). Regardless of the system used, persisting organ failure indicates a severe attack. A serum C-reactive protein level >150 mg/L at 48 hours after the onset of symptoms is also an indicator of severity. Patients with a body mass index over 30 are at higher risk of developing complications. A revision in 2013 of the Atlanta classification of acute pancreatitis (1992) recommends that patients with acute pancreatitis be stratified into 3 groups:

- Mild acute pancreatitis:
- no organ failure;
- no local or systemic complications.
- Moderately severe acute pancreatitis:
- organ failure that resolves within 48 hours (transient organ failure); and/or
- local or systemic complications without persistent organ failure.
- Severe acute pancreatitis:
- persistent organ failure (>48 hours);
- single organ failure;
- multiple organ failure.

ChatGPT:

Which of the following is the most common cause of acute pancreatitis?

A) Biliary calculi

B) Alcohol abuse

C) Tumours at the ampulla of Vater

D) Hereditary pancreatitis

Answer: A) Biliary calculi
